# Intracranial pressure management in patients with human immunodeficiency virus-associated cryptococcal meningitis in a resource-constrained setting

**DOI:** 10.4102/sajhivmed.v21i1.1171

**Published:** 2020-12-18

**Authors:** Philasande Mkoko, Jessica Du Preez, Senlika Naidoo

**Affiliations:** 1 Department of Medicine, Division of Cardiology, Faculty of Health Sciences, University of Cape Town, Cape Town, South Africa; 2 Department of Medicine, Dora Nginza Hospital, Port Elizabeth, South Africa; 3 Department of Medicine, Livingstone Hospital, Port Elizabeth, South Africa

**Keywords:** cryptococcal meningitis, HIV, antifungal therapy, antiretroviral therapy, in-hospital mortality, adult PLWH

## Abstract

**Background:**

Cryptococcal meningitis (CCM) is the leading cause of meningitis in people living with HIV (PLWH) in sub-Saharan Africa (SSA). The mortality and morbidity associated with CCM remain high. Combination of antifungal therapy, diligent management of intracranial pressure (IP) and the correct timing of the introduction of antiretroviral therapy (ART) minimise the risk of mortality and morbidity. The absence of spinal manometers in many healthcare centres in SSA challenges the accurate measurement of cerebrospinal fluid (CSF) pressure and its control.

**Objectives:**

We hypothesised that four lumbar punctures (LPs) in the first week of the diagnosis and treatment of CCM would reduce IP such that in-hospital mortality and morbidity of HIV-associated CCM (HIV/CCM) would be significantly reduced.

**Methods:**

We conducted a retrospective study to assess whether receipt of four or more LPs in the first week of the diagnosis and treatment with combination antifungal therapy of HIV/CCM would be associated with the reduction of in-hospital mortality in adult PLWH.

**Results:**

From 01 January 2016 to 31 December 2016, 116 adult patients were admitted to the Dora Nginza District Hospital in Zwide, Port Elizabeth, South Africa. After exclusion of 11 (two were younger than 18 years, two had missing hospital records and seven demised or left the hospital before 7 days of hospitalisation), 105 patients were included in the analysis. The mean age was 39.4 (standard deviation [s.d.] ± 9.7) years, 64.8% were male. All were PLWH. A total of 52.4% had defaulted ART and 25.7% were ART naïve. Forty-three patients received four or more LPs (mean = 4.58 [± 0.96]) in the first week of hospitalisation with an associated in-hospital mortality of 11.6% (*n* = 5/43) compared with 62 patients who received less than four LPs (mean = 2.18 [± 0.80]) with an in-hospital mortality of 29% (*n* = 18/62) and a relative risk of 0.80 (95% CI, 0.66–0.97), *p* = 0.034.

**Conclusion:**

In the current study of adult PLWH presenting to hospital with HIV/CCM, four or more LPs in the first 7 days following admission and the initiation of treatment were associated with a 17.4% reduction in absolute risk of in-hospital mortality and a 20% reduction in relative risk of in-hospital mortality. This mortality difference was noted in patients who survived and were in hospital at the time of the 7-day study census and persisted until the time of hospital discharge.

## Background

Cryptococcal meningitis (CCM) accounts for up to 60% of meningitis in adult persons living with HIV (PLWH) in many African countries including South Africa (SA).^1,2^ Those with CD4 cell counts < 100 cells/µL are particularly at risk.^3^ Mortality is high – reaching levels of 70% in sub-Saharan Africa (SSA).^3^ Altered mental state at presentation, older age, high cerebrospinal fluid (CSF) fungal burden and high peripheral white cell count predict mortality in antiretroviral therapy (ART) naïve patients.^4^ Although the availability of ART has led to a decrease in HIV-associated CCM (HIV/CCM) in high-income countries,^5^ the condition remains responsible for 10% – 20% of HIV-related deaths in SSA.^6^ Notwithstanding improved access to ART, many remain outside of care or on failing treatment and at risk of opportunistic disease.^7^

The initial (induction phase) management of HIV/CCM requires the following: (1) combination antifungal therapy including IV amphotericin B and oral flucytosine (first week only) and high-dose oral fluconazole 1200 mg daily (second week), after that an 8-week consolidation phase of oral fluconazole 800 mg daily, (2) control of raised intracranial pressure (rIP) with therapeutic lumbar punctures (LPs) to maintain the ‘opening-pressure’ (CSF-OP) at < 25 cm of water and (3) minimising the risk of immune reconstitution inflammatory syndrome (IRIS) by delaying the initiation of ART until 4 to 6 weeks after the start of antifungal therapy.^8,9,10,11,12^

In the absence of a spinal manometer, the SA guidelines for the prevention, diagnosis and management of CCM recommend performing an LP to remove 20 mL – 30 mL of CSF if the symptoms and signs of rIP are present.^12^ However, in clinical practise only 23% – 30% of CCM patients with signs and symptoms receive ‘therapeutic’ LPs.^13,14^ A symptom guided approach has the potential to miss asymptomatic patients who might benefit from therapeutic LPs.

We hypothesised that four or more LPs in the first 7 days of treatment could facilitate CSF drainage and reduce in-hospital mortality in PLWH and HIV/CCM in a resource constrained setting where there are no spinal manometers. We, therefore, conducted a single centre retrospective cohort study to determine the impact on in-hospital mortality of four or more LPs in the first 7 days of antifungal therapy compared with PLWH/CCM who received fewer than four LPs.

## Methods

### Study design

The study was designed as a retrospective cohort review of PLWH/CCM admitted to the department of medicine at the Dora Nginza Hospital from 01 January 2016 to 31 December 2016. The Dora Nginza Hospital is a district hospital located in the Zwide township of the Nelson Mandela Bay Municipality (Port Elizabeth), SA. The Nelson Mandela Bay region has a population of 1 152 115 and an unemployment rate of 36.6%.^15^ The internal medicine department consists of a 120-bed unit *without* access to intensive or high care services and with limited access to radiological imaging apart from plain chest radiography.

### Study population

Clinical notes, discharge summaries and death notification registries were reviewed to identify patients who received a primary or secondary diagnosis of CCM.

### Data collection

A standardised data collection form was prepared. This included patient demographic details, comorbidities, history of previous CCM, data of concurrent tuberculosis (TB) and details of the index admission. Patients’ folders were checked for the results of therapeutic LPs and to document the indications for the procedure. The National Health Laboratory Services (NHLS) computer records were accessed for admission bloods, CD4 count, HIV viral load (VL) and CSF results. Identifying patient material was anonymised at the time of the collection and storage of data.

### Statistical analysis

Continuous variables are reported as mean (± standard deviation, [s.d.]) when normally distributed and as median (interquartile range, IQR) when not normally distributed. Discrete data are presented as number and percentages. Pearson’s chi-square test and corresponding 95% Confidence Intervals (CI) were used to calculate the mortality difference between the study and comparator groups. A *p*-value of < 0.05 defined significance. Patients who died or left hospital *within the first 7 days* were excluded from the mortality analysis. Statistical analyses were performed by using IBM SPSS Statistics for Macintosh version 24.0.

### Ethical consideration

Approval to conduct the study was obtained from Walter Sisulu University Human Research Committee. Ethical clearance number: 027/2018.

## Results

### Clinical characteristics of the study population

**FIGURE 1:**
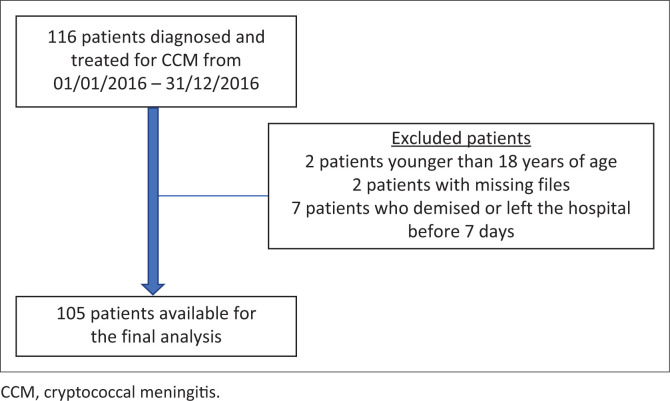
Flow diagram showing patients enrollment in the study.

From 01 January 2016 to 31 December 2016, a total of 116 patients received a diagnosis of CCM. After exclusion of 11 (two were younger than 18 years, two had missing hospital files/records and seven demised or left the hospital before 7 days of hospitalisation), 105 patients were available for study analysis (Figure 1).

The mean age of patients was 39.4 (s.d. ± 9.7) years. A total of 65.2% were male. All the patients were PLWH and had a median CD4 count = 37 (IQR, 13–77) cells/µL. Human immunodeficiency virus infection was newly diagnosed on the index admission in 25.7%. Default from previous ART was recorded in 52.4% of patients (Table 1).

**TABLE 1:** Baseline characteristics.

Demographic data	Overall *N* = 105					
	Mean	s.d.	Median	IQR	*n*	%
Age in years	39.4	9.7	-	-	-	
Male – No	-	-	-	-	68	64.8
HIV infected – No	-	-	-	-	105	100
Newly diagnosed HIV – No	-	-	-	-	27	25.7
Defaulted ART – No	-	-	-	-	55	52.4
ART naïve – No	-	-	-	-	27	25.7
1st line ART – No	-	-	-	-	22	21.0
2nd line ART – No	-	-	-	-	1	1.0
CD4 count – cells/µL	-	-	37	13–77	-	-
HIV viral load – copies/mL	-	-	146 132	46 099–501 761	-	-
Undetectable HIV viral load – No	-	-	-	-	4	3.8
Previous CCM – No	-	-	-	-	18	17.1

s.d., standard deviation; HIV, human immunodeficiency virus; ART, antiretroviral therapy; IQR, interquartile range; CCM, cryptococcal meningitis.

A total of 17.1% of patients gave a history of previous CCM. Headache was the most prevalent presenting symptom (91.4%) followed by a low Glasgow Coma Scale (32.4%) (Table 2). A total of 9.5% of patients had a focal neurological deficit, namely an abducent (cranial nerve 6) palsy, suggesting possible rIP at presentation. The diagnosis of CCM was based on a positive CSF-Cryptococcal Antigen (CrAg) in all patients, a positive CSF India Ink in 80% and a positive CSF culture for *Cryptococcus neoformans* in 94.3%. One hundred and three (*n* = 103) patients received local guideline-based therapy consisting of a combination of amphotericin B and fluconazole. Two patients did not receive fluconazole: one had acute hepatitis B and the other, a drug-induced liver injury (DILI) resulting from TB therapy.

**TABLE 2:** Clinical features and cerebrospinal fluid findings.

Variable	*N* = 105					
	Mean	s.d.	Median	IQR	*n*	%
Positive screening serum CrAg – No	-	-	-	-	9	8.6
Headache – No	-	-	-	-	96	91.4
Low GCS – No	-	-	-	-	34	32.4
Seizures – No	-	-	-	-	5	4.8
Focal neurology – No	-	-	-	-	10	9.5
Skin rash – No	-	-	-	-	17	16.2
Cerebrospinal fluid analysis	-	-	-	-	-	-
Protein – Mean (s.d.) g/L	1.15	1.0	-	-	-	-
Glucose – Mean (s.d.) mmol/L	2.40	1.50	-	-	-	-
Lymphocytes – Median (IQR)/µL	-	-	9.0	2–64	-	-
Polymorphonuclear cells – Median (IQR)/µL	-	-	3.00	0–9.0	-	-
Positive CrAg – No	-	-	-	-	105	100
Positive India Ink – No	-	-	-	-	84	80.0
Positive culture for cryptococcus neoformans – No	-	-	-	-	99	94.3
In-hospital therapy	-	-	-	-	-	-
Amphotericin B plus fluconazole – No	-	-	-	-	103	98.1
Amphotericin B only – No	-	-	-	-	2	1.9
Therapeutic lumbar punctures	4.62	2.30	-	-	-	-
Number of days in hospital	19.4	8.3	-	-	-	-
In-hospital mortality – No	-	-	-	-	23	21.9

CrAg, cryptococcal antigen; GCS, GCS, Glasgow Coma Scale; s.d., standard deviation; IQR, interquartile range.

### Therapeutic lumbar punctures and hospital mortality

A total of 496 LPs were performed. Each patient received a mean of 4.62 (s.d. ± 2.30) therapeutic LPs (all patients). The mean duration of hospitalisation of the entire group was 19.4 (s.d. ± 8.3) days. A total of *n* = 23/105 (21.9%) patients died during the index hospitalisation.

**FIGURE 2:**
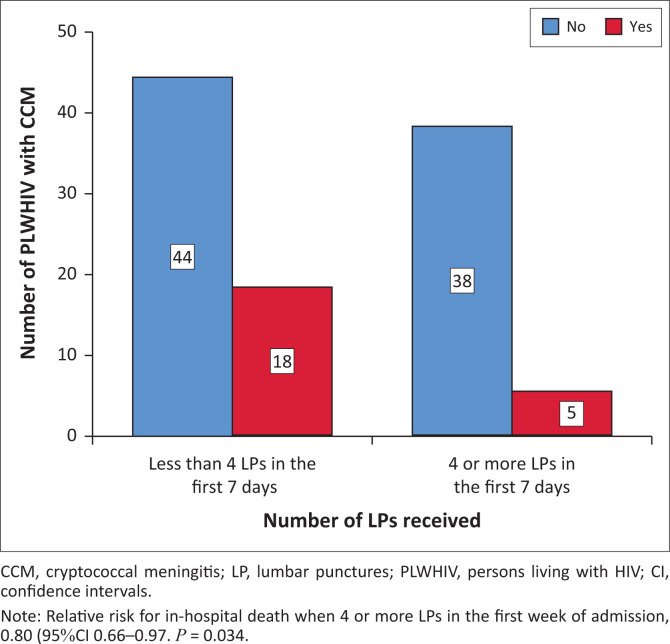
Clustered bar chart depicting in-hospital mortality and receipt of ≥ 4 lumbar punctures versus < 4 lumbar punctures in the first 7 days of combination antifungal therapy.

**FIGURE 3:**
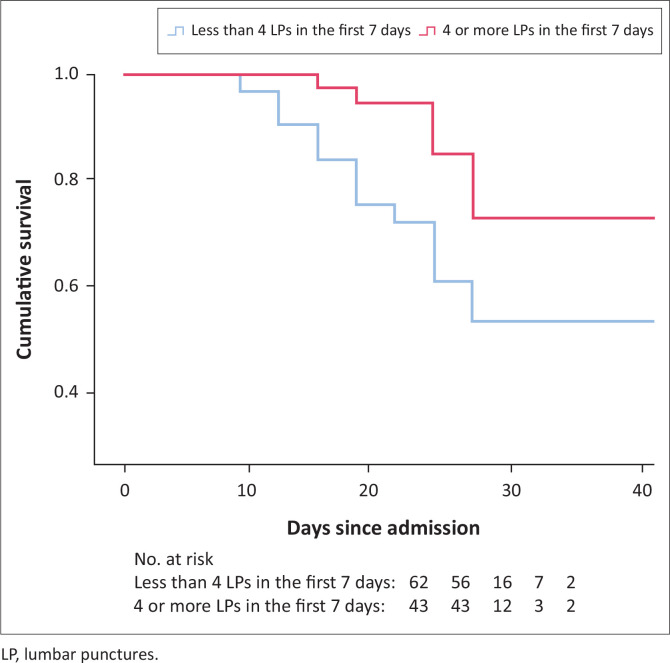
Kaplan-Meier estimate of survival amongst patients who received four or more lumbar punctures in the first 7 days of combination antifungal therapy compared with those who received less than four lumbar punctures in the first 7 days of combination antifungal therapy.

Patients who received ≥ 4 LPs in the first 7 days had an in-hospital mortality rate of 11.6% (*n* = 5/43), whereas those with < 4 LPs in the first 7 days had in-hospital mortality of 29% (*n* = 18/62). This represents a 17.4% absolute risk reduction of in-hospital mortality and a relative risk of 0.80 (95% CI, 0.66–0.97, *p* = 0.034), namely a 20% relative risk reduction of in-hospital mortality (Figures 2 and 3). Patients who received four or more LPs in the first 7 days received a mean of 4.58 (s.d. ± 0.96) LPs in the first week of treatment.

## Discussion

In this retrospective study of patients with HIV/CCM, receipt of four more LPs in the first week of diagnosis and treatment was associated with reduced in-hospital mortality. These findings inform the recorded 98.2% compliance of hospital staff with local guideline-based treatment of CCM with combination antifungal therapy.

Raised intracranial pressure develops in most PLWH with HIV/CCM and portends a poor prognosis if not adequately treated.^16^ Lumbar punctures and CSF drainage have been shown to be effective in managing CCM related rIP.^17^ Alternatives such as acetazolamide or corticosteroids have no role in the management of HIV/CCM.^18,19^ Despite the increased prevalence of rIP, therapeutic LPs are seldom instituted even when symptoms and signs of rIP are present.^13,16^ In a clinical audit by Adeyemi and Ross, only 23% of patients with CCM related headaches received therapeutic LPs despite 82% of patients receiving analgesia for their pain.^13^ Similarly, Rolfes et al. report that only 30% of the 248 patients in their cohort received therapeutic LPs.^14^ This was despite the fact that therapeutic LPs were associated with a 69% improvement in survival.^14^ In our study we report a 17.4% absolute risk reduction of in-hospital mortality following intervention with four or more LPs in the week of diagnosis and treatment.

Spinal manometers are recommended for the measurement of rIP. In resource limited settings spinal manometers are seldom available. Instead, guidelines recommend using tubing from intravenous giving-sets.^20^ A small single centre study by Meda and colleagues has confirmed a correlation between spinal manometer and intravenous giving set use in determining CSF-OP in the setting of CCM. However, this study consisted of only 35 subjects and reported technical shortcomings in the reliability of the measurements.^21^ In a recent study, Mogambery et al. found that the use of an intravenous giving set considerably underestimated CSF-OP when compared with that of a spinal manometer, mean 16.2 (s.d. ± 10) cm H_2_O versus 22.7 (s.d ± 10) cm H_2_O, *p* < 0.001.^22^ A schedule of at least four LPs with CSF drainage of 20 mL – 30 mL^12^ in the first week of diagnosis and treatment could be life-saving in settings with no access to spinal manometers.

The optimal management of CCM consists of a triad of (1) combination antifungal therapy, (2) intracranial pressure (IP) management with CSF drainage and (3) immune reconstitution with ART after completion of 4 to 6 weeks of combination antifungal therapy to avoid CCM-IRIS.^12^ This study provides evidence that rIP in HIV/CCM can be managed without recourse to spinal manometers.

The limitations of this study include it’s retrospective and single centres design. Also, we do not have data on the volume of CSF removed and survival beyond the index hospitalisation.

In conclusion, this study shows that PLWH/CCM can be effectively managed in centres with limited access to spinal manometers. We have shown that ≥ four LPs with CSF drainage in the first 7 days of hospitalisation improves early survival.
